# FKBP5 as a Selection Biomarker for Gemcitabine and Akt Inhibitors in Treatment of Pancreatic Cancer

**DOI:** 10.1371/journal.pone.0036252

**Published:** 2012-05-09

**Authors:** Junmei Hou, Liewei Wang

**Affiliations:** Division of Clinical Pharmacology, Department of Molecular Pharmacology and Experimental Therapeutics, Mayo Clinic, Rochester, Minnesota, United States of America; Technische Universität München, Germany

## Abstract

We have recently shown that the immunophilin FKBP5 (also known as FKBP51) is a scaffolding protein that can enhance PHLPP-AKT interaction and facilitate PHLPP-mediated dephosphorylation of Akt Ser473, negatively regulating Akt activation *in vitro*. Therefore, FKBP5 might function as a tumor suppressor, and levels of FKBP5 would affect cell response to chemotherapy. In the current study, we have taken a step forward by using a pancreatic cancer xenograft mice model to show that down regulation of FKBP5 in shFKBP5 xenograft mice promotes tumor growth and resistance to gemcitabine, a phenomenon consistent with our previous findings in pancreatic cell lines. In addition, we also found that inhibitors targeting the Akt pathway, including PI3K inhibitor, Akt inhibitor and mTOR inhibitor had a different effect on sensitization to gemcitabine and other chemotherapeutic agents in cell lines, with a specific Akt inhibitor, triciribine, having the greatest sensitization effect. We then tested the hypothesis that addition of triciribine can sensitize gemcitabine treatment, especially in shFKBP5 pancreatic cancer xenograft mice. We found that combination treatment with gemcitabine and triciribine has a better effect on tumor inhibition than either drug alone (p<0.005) and that the inhibition effect is more significant in shFKBP5 xenograft mice than wt mice (p<0.05). These effects were correlated with level of Akt 473 phosphorylation as well as proliferation rate, as indicated by Ki67 staining in xenograft tumor tissues. These results provide evidence in support of future clinical trials designed to tailor therapy based on our observations.

## Introduction

Cytidine analogues such as gemcitabine are widely used to treat a variety of cancers. Gemcitabine remains standard therapy for pancreatic cancer in the adjuvant and palliative settings [1], [2], [3]. However, the gemcitabine response rate is very low in pancreatic cancer, with only an 18% 1 year survival rate [4]. This poor survival rate is primarily because of the lack of early detection and frequent metastasis of primary tumors into lymph nodes and surrounding organs, such as the liver and stomach [5], [6], [7]. As a step toward individualized gemcitabine therapy in order to achieve better outcomes, we previously performed a genome wide association study using 197 individual lymphoblastoid cell lines [8], [9] and identified a protein, FKBP5, that showed a significant effect on gemcitabine response in tumor cells by negatively regulating Akt phosphorylation at serine 473 [10], [11]. Phosphorylation of Akt activates the Akt pathway, which plays a critical role in tumorigenesis and chemoresistance [12], [13], [14], [15]. Therefore, low FKBP5 expression renders tumor cells resistant to many chemotherapeutic agents, including gemcitabine [8]. In addition, FKBP5 expression is low or lost in many pancreatic cancer cell lines and pancreatic cancer patient samples, correlating with increased Akt Ser473 phosphorylation [10].

These results suggested that FKBP5 might be a tumor suppressor and that levels of FKBP5 might determine patients’ response to chemotherapy. If that is correct, patients with low levels of FKBP5 and Akt hyperactivation might benefit from the addition of inhibitors targeting the Akt pathway. In the current study, we tested that hypothesis by using an FKBP5 knockdown pancreatic cancer xenograft mouse model (shFKBP5) and the results of these experiments may form a foundation for future clinical translational studies. We found that shFKBP5 xenograft mice showed a significant increase in tumor burden compared with wtFKBP5, and that these tumors were more resistant to gemcitabine treatment. While both wt and shFKBP5 xenograft mice were able to benefit from combination therapy with gemcitabine and the Akt inhibitor, triciribine (TCN), shFKBP5 mice showed a greater effect after combination treatment.

## Materials and Methods

### Cell Lines

The human pancreatic cancer cell lines SU86, ASPC1, and BXPC3 were gifts from Dr. Daniel D. Billadeau, Mayo Clinic. Human breast cancer cell lines HS578T (HTB-126™) and MCF7 (HTB-22™) were obtained from the American Type Culture Collection (ATCC). The pancreatic cancer cell lines were cultured in RPMI 1640 supplemented with 10% fetal bovine serum, and maintained in an incubator with a humidified atmosphere of 5% CO_2_ at 37°C. The human breast cancer cells were cultured with DMEM supplemented with 10% fetal bovine serum under the conditions described above.

### Plasmids and Short Interfering RNA

The FKBP5 shRNA and siRNA used in the knock down studies were purchased from QIAGEN Inc. (Valencia, CA). Transfection was performed twice, 24 hours apart, with 200 nM siRNA using the Lipofectamine™ RNAiMAX reagent (Invitrogen, Carlsbad, CA) according to the manufacturer’s instructions.

Sequences for shRNA against FKBP5 were:

First FKBP5 shRNA.

Sense strand: GGG UAA ACA GAU UGA GCA UdTdT.

Antisense strand: AUG CUC AAU CUG UUU ACC CdGdT.

Second FKBP5 shRNA.

Sense strand: AAU AUC CCU CUC CUU UCC GdTdT.

Antisense strand: CGG AAA GGA GAG GGA UAU UdGdT.

Both FKBP5 shRNA duplexes were cloned into pSuper vectors, and pooled for transfection. The packaging cell line 293T was infected with retrovirus pSuper–shRNA [16]. The medium was changed 24 hours later and collected 48 or 72 hours after transfection. The medium was then filtered through sterile filters (0.45–µm filter) and was used to infect SU86 cells. Infected cells were selected with 2 µg/ml puromycin (Sigma-Aldrich). Sixteen puromycin -resistant colonies that were verified by RT-PCR were then pooled and stable transfectants were maintained in RPMI 1640 supplemented with 10% FBS and 2 µg/ml puromycin.

Sequences for siRNA against FKBP5 were:

Sense strand: GGG UAA ACA GAU UGA GCA UdTdT.

Antisense strand: AUG CUC AAU CUG UUU ACC CdGdT.

Sequences for negative control siRNA were:

Sense strand: UUC UCC GAA CGU GUC ACG UdTdT.

Antisense strand: ACG UGA CAC GUU CGG AGA AdTdT

### Drugs and Cell Proliferation Assays

Gemcitabine was provided by Eli Lilly (Indianapolis, IN). Triciribine (TCN), rapamycin and LY 294002 were purchased from EMD Biosciences (San Diego, CA). Etoposide and paclitaxel were purchased from Sigma-Aldrich (St. Louis, MO). Cytotoxicity assays with the tumor cell lines were performed with the CellTiter 96® AQueous Non-Radioactive Cell Proliferation Assay (Promega Corporation, Madison, WI) as described previously [17]. Cytotoxicity was assessed by plotting cell survival versus drug concentration (on a log scale). The IC50 phenotype (effective dose that kills 50% of the cells) was calculated using a four parameter logistic model and was used for statistical comparison between different treatments.

The growth-inhibitory effects of gemcitabine, etoposide and paclitaxel as well as the effects of combination treatment with TCN, rapamycin and LY 294002 in BXPC3, ASPC1, SU86, MCF7 and HS578T cells were also determined using the MTS assay. Results reported represent the averages of three independent replicates.

### Transient Transfection and RNA Interference

Human BXPC3 and ASPC1 pancreatic cancer cell lines as well as MCF7 and HS578T breast cancer cell lines were used to perform the siRNA studies. The Hiperfect transfection reagent (QIAGEN) was used for siRNA reverse transfection. Specifically, cells were seeded into 96-well plates and were mixed with siRNA-complex, consisting of 10 nM of specific or negative control siRNA (QIAGEN) and 0.1 µl of lipofectamine™ RNAiMAX reagent (Invitrogen, Carlsbad, CA).

### Quantitative Real-time Reverse Transcription PCR

Total RNA was isolated from cultured cells with the Qiagen RNeasy kit (QIAGEN Inc. Valencia, CA), followed by QRT-PCR performed with the 1-step, Brilliant SYBR Green QRT-PCR master mix kit (Stratagene, La Jolla, CA). Specifically, primers purchased from Qiagen were used to perform QRT-PCR using the Stratagene Mx3005P™ Real-Time PCR detection system (Stratagene). All experiments were performed in triplicate with β-actin as an internal control. Reverse transcribed Universal Human reference RNA (Stratagene) was used to generate a standard curve. Control reactions lacked RNA template.

### Western Blot Analyses

SDS-PAGE and Western blot analysis were carried out as previously described [10]. FKBP5 antibodies were raised against GST fusion proteins containing amino-terminal residues 1–100 of FKBP5. Antibodies against Akt (#9272), phospho-Akt (Ser473) (#9271), phospho-Akt (Thr308), FOXO1 (#9454), phospho-FOXO1 (Thr 24) (#9464), GSK3β (#9315) and phospho-GSK3β (#9316) were purchased from Cell Signaling Inc (Boston, MA). Tumor specimens were processed for Western blotting as described previously using a Triton X-100-containing lysis buffer [10]. Blots were developed with Super Signal Chemiluminescence reagent (Pierce, Rockford, IL).

### Athymic Nude Mouse Tumor Formation Assay

All mice used in this study were maintained in the Mayo Clinic Animal Breeding Facility. All experimental protocols were reviewed and approved by the Mayo Clinic Institutional Animal Care and Use Committee (protocol no. A14008), and all studies were performed according to the methods approved in the protocol.

SU86 cells stably expressing FKBP5 shRNA and mock cells were injected subcutaneously into the left inguinal area of 4-week-old female athymic recessive nude/nude mice (athymic Ncr-nu/nu: National Cancer Institute-Frederick) using 19-gauge needles [18], [19]. Each mouse received one injection of 5×10^6^ cells in 200 µl serum-free DMEM. Animals were monitored for activity and physical condition every day, and the determinations of body weight and measurement of tumor mass were performed every 3 days. Tumor growth was monitored for 6 weeks, and tumor volume was calculated as 1/2a × b^2^, where “a” stands for the long diameter and “b” is the short diameter [18], [19]. The tumors were then surgically removed and processed.

### Therapy Evaluation

Mice bearing subcutaneous wtFKBP5 SU86 and shFKBP5 SU86 tumors entered the study when tumors reached ∼100 mm^3^ (day 0) and were randomized to treatment groups, with 5 mice in each group. Gemcitabine was administered i.p. every 3 days at concentrations of 25, 50 or 100 mg/kg. For the gemcitabine/TCN study, four treatment groups were included: (a) vehicle, (b) TCN, at a dose of 0.5 mg/kg/d in 1.5% sodium bicarbonate (Thermo Fisher) w/v in water, pH 8.0, administered in 100 mL intraperitoneal injections once daily from Monday to Friday for 4 weeks, (c) gemcitabine, at a dose of 50 mg/kg in saline, administered i.p. every 3 days for 4 weeks, or (d) a combination of the two treatments. There was no evidence of gross toxicity in the drug-treated animals as measured by weight loss. The tumor growth rate was calculated with the size measured at each time point normalized to the initial tumor volume at day 0 when tumors of shFBKP5 and wtFKBP5 xenograft mice reached 100 mm^3^. Results of the treatment effect were represented by tumor inhibition ratio, defined as tumor growth rate of shFKPB5 mice corrected for that of wt FKBP5 mice. Maximal suppression of tumor growth was used for statistical comparison between different treatment groups.

### Immunohistochemical Staining

The tissue sections were deparaffinized in xylene, dipped in decreasing concentrations of ethyl alcohol, and then rehydrated in distilled water. Antigen retrieval for Ki67 (Abcam, Cambridge, MA) was performed by placing slides in preheated EDTA as the retrieval solution in a steamer at 98°C for 30 minutes. The staining procedure was carried out in a Dako Autostainer Plus. Specifically, the tissue sections were treated with Peroxidase Blocking Reagent (Dako, Carpinteria, CA) for 5 minutes and then were washed with 1x Wash Buffer (Dako, Carpinteria, CA), followed by treatment with Protein Block Serum-Free (Dako, Carpinteria, CA) for 5 minutes. The tissue sections were then incubated with the Ki67 primary antibody for 60 minutes at room temperature, followed by incubation with the secondary antibody (Dako, Carpinteria, CA) for 15 minutes. High-sensitivity diaminobenzidine (DAB+) chromogenic substrate system (Dako, Carpinteria, CA) was used for colorimetric visualization.

### Statistical Analysis

The experimental data are expressed as mean ± SEM. Differences between control and treated groups were determined by the use of paired *t* test or ANOVA, and p<0.01 was considered to be statistically significant.

## Results

### Knockdown of FKBP5 Results in Increased Pancreatic Tumor Growth and Gemcitabine Resistance

**Figure 1:**
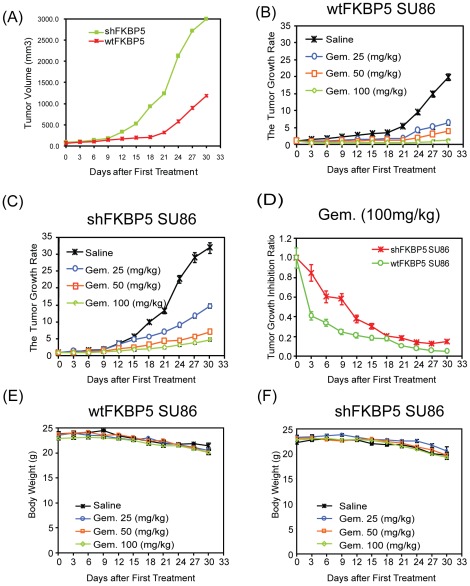
Loss of FKBP5 expression in pancreatic cancer cells results in increased tumor growth in mice. Mice bearing subcutaneous wtFKBP5 SU86 and shFKBP5 SU86 tumors entered the study when tumors reached ∼100 mm^3^ (day 0). All mice were randomized to control or treatment groups and were treated with vehicle or gemcitabine at doses of 25, 50, and 100 mg/kg intraperitoneally twice per week. (A) Formation of subcutaneous (s.c.) tumors in wt and shFKBP5 SU86 injected nude mice. Results are represented by the tumor volume measured at each time point. **P<0.001 (5 mice/group) as compared between wtFKBP5 and shFKBP5 xenografts treated with saline. (B and C) Gemcitabine response in wt or shFKBP5 xenograft mice. Results are represented by the tumor volume measured at each time point corrected to day 0 when mice entered the study. (D) shFKBP5 xenografts are resistant to gemcitabine *in vivo*. Results are represented by tumor inhibition ratio, defined as the measurements of tumor volume at each time point normalized to day 0 for shFKPB5 mice, corrected for that of wt FKBP5 mice. **P<0.001; *P<0.05 (5 mice/group) as a comparison of tumor growth inhibition ratio between wtFKBP5 and shFKBP5 treated with 100 mg/kg of gemcitabine. (E and F) Measurements of body weight for wt and shFKBP5 xenografts. Body weight was recorded for each mouse every 3 days throughout the experiment. Statistical significance was assessed by two-way ANOVA.

Previous studies have demonstrated that FKBP5 expression is down-regulated in pancreatic cancer and have suggested that FKBP5 may be involved in the tumorigenesis of pancreatic cancer. The SU86 pancreatic cancer cell line was stably transfected with pooled FKBP5 shRNA. We then determined the effect of FKBP5 on the formation of xenograft tumors. There was a dramatic increase of tumor size in FKBP5 knockdown mice compared with control mice, indicating that FKBP5 is a potential tumor suppressor (Figure 1A). As shown in Figure 1A, the tumor volume was significantly greater in shFKBP5 mice than in control mice. At day 18, the mean volume was 200±101 mm^3^ in control animals (n = 5 mice/group), and 937±103 mm^3^ in shFKBP5 mice (n = 5; p<0.001). This trend was consistent until day 30 when the mice were sacrificed (shFKBP5 mice: 2999±298 mm^3^, and wtFKBP5 mice: 1190±243 mm^3^; n = 5; p<0.001). Since our previous studies showed that the expression level of FKBP5 was correlated with the sensitivity of pancreatic cancer cells to chemotherapeutic drugs [10], we next determined whether knockdown of FKBP5 could affect the chemosensitivity of SU86 xenografts to gemcitabine *in vivo*. We first tested the dose effect of gemcitabine with both wt and shFKBP5 SU86 xenografts once tumors reached the same size, 100 mm^3^. A dose-dependent inhibition of tumor growth was observed with gemcitabine for all the SU86 xenografts (Figure 1B and C). FKBP5 wild type SU86 xenografts showed a statistically significant response to 100 mg/kg of gemcitabine treatment compared with shFKBP5 SU86 xenografts treated with the same dose of gemcitabine (Figure 1D, p<0.05), suggesting that low expression of FKBP5 can cause resistance to gemcitabine. We also found that at the lower concentrations of gemcitabine, the wtFKBP5 also exhibited a trend toward better response than shFKBP5 xenograft mice, although not statistically significant (data not shown). All treatments were well tolerated, with no significant body weight loss (Figure 1E and F).

**Figure 2:**
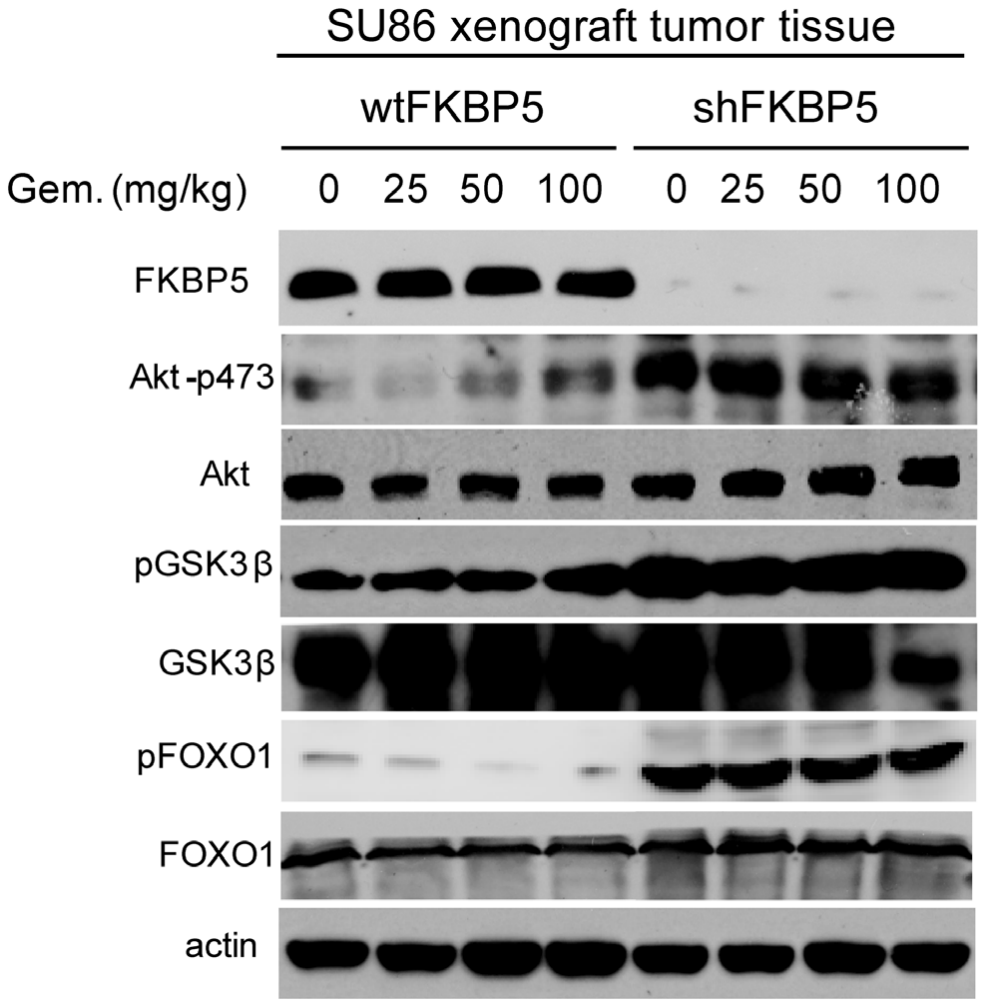
FKBP5 Regulates Akt Phosphorylation at Ser473 *in vivo*. Tumor samples from wtFKBP5 SU86 or shFKBP5 SU86 xenografts were prepared and analyzed for phosphorylation of Akt and downstream signaling molecules by Western blot analysis.

We have previously shown that activated Akt signaling is associated with low levels of FKBP5 in pancreatic cancer cells [10]. Therefore, we examined the activity of the Akt pathway in tumor samples for each cell line. In shFKBP5 xenografts, phosphorylated Akt-Ser473, FOXO1and GSK3β were significantly increased compared with the control (Figure 2, p<0.01). Addition of gemcitabine had no effect on levels of phosphorylation for these proteins. These results were consistent with our previous findings using pancreatic cell lines [10]. Collectively, this series of experiments suggests that FKBP5 functions as a tumor suppressor by negatively regulating the Akt pathway *in vivo*. In addition, the level of FKBP5 affects sensitivity to gemcitabine treatment associated with its effect on Akt phosphorylation in the pancreatic xenograft model.

### Akt Inhibitor Sensitizes Tumor Cells with Low FKBP5 to Chemotherapeutic Agents *in vitro*

The phosphatidylinositol 3-kinase (PI3K)/Akt pathway is a cell survival pathway that is important for normal cell growth and proliferation [20], [21], [22], [23]. This pathway is also an important target for cancer treatment, including mammalian target of rapamycin (mTOR) inhibitors, inhibitors of PI3K and inhibitors of Akt that have already demonstrated clinical efficacy for different tumors [24]. Since FKBP5 negatively regulates Akt activity, we would expect that the addition of inhibitors targeting the Akt pathway might reverse resistance to gemcitabine. To test this hypothesis, we performed a series of *in vitro* experiments using three pancreatic tumor cell lines (ASPC1, BXPC3 and SU86) and two breast cancer cell lines (MCF7 and HS578T). We selected three different Akt pathway inhibitors, including an upstream inhibitor of PI3K, LY294002, a specific Akt inhibitor, triciribine (TCN) that inhibits phosphorylation of all three isoforms of Akt, and an mTOR inhibitor, rapamycin. We then evaluated the cytotoxicity effect of gemcitabine in combination with LY294002, TCN, and rapamycin, respectively. Table 1 summarizes IC50 values of each treatment for these five cell lines. Our data confirmed, once again, that knockdown of FKBP5 desensitized cells to gemcitabine treatment in all of the cell lines tested (Table 1 and Figure S1). LY294002, TCN and rapamycin had very modest effects when used alone in either FKBP5 knockdown cells or control cells, especially at the concentrations (10 µM of TCN, 1.4 µM LY294002, and 1 nM rapamysin) that we used for combination treatments (Figure S2). TCN sensitized both control and FKBP5 knockdown cells to gemcitabine (Table 1, and , p<0.005). However, the TCN sensitization effect was greater in FKBP5 knockdown cells than in wtFKBP5 cells (p<0.001) (Table 1 and Figure S1). The sensitization effects of LY294002 and rapamycin were much less than that of TCN (Table 1 LY294002, p  = 0.0023∼0.3412; rapamycin, p  = 0.0171∼0.931).

**Table 1:** Combinatory effects of gemcitabine and inhibitors targeting PI3K-Akt-mTOR pathway in human pancreatic and breast cancer cells.

Cells	Agent	IC_50_ (nM)^a^		p value^b^	
		Neg.siRNA	siFKBP5	Neg.siRNA	siFKBP5
BXPC3	Gem	7.45	17.53		
	Gem+TCN	1.179	2.634	0.0005	0.0003
	Gem+LY294002	2.842	6.839	0.0023	0.001
	Gem+Rap	9.956	12.19	0.1509	0.1755
					
ASPC1	Gem	4.935	15.48		
	Gem+TCN	1.725	4.977	0.001	0.0007
	Gem+LY294002	3.122	8.343	0.1985	0.0216
	Gem+Rap	3.356	13.74	0.0171	0.2744
					
MCF7	Gem	5.697	15.63		
	Gem+TCN	1.999	2.925	0.0036	0.0003
	Gem+LY294002	4.339	8.509	0.0045	0.0054
	Gem+Rap	5.369	10.84	0.9096	0.0769
					
HS578T	Gem	3.447	14.58		
	Gem+TCN	1.115	2.92	0.003	0.001
	Gem+LY294002	4.095	8.177	0.3412	0.0099
	Gem+Rap	3.332	9.903	0.931	0.0342

a The values represent the average of three independent experiments.

b IC50 values between combination treatment vs. Gem along were analyzed statistically by performing t tests.

Abbreviations: Gem, gemcitabine; TCN, tricirbine; Rap, rapamycin.

We had previously found that level of FKBP5 also affects response to other chemotherapeutic agents, including etoposide and taxanes [10]. Therefore, we tested whether TCN could also sensitize those agents in the four cell lines studied. In all four cell lines, FKBP5 knockdown made the cells more resistant to etoposide treatment alone, which is consistent with previous findings. We found that TCN could significantly sensitize etoposide in BXPC3, ASPC1, HS578T and MCF7 cells when compared IC50 values for etoposide treatment alone vs. different combination treatments (Table S1). The sensitization effect was more prominent in cells with FKBP5 knockdown. LY294002 could also sensitize etoposide in BXPC3 and MCF7 cells with both control and siFKBP5 transfection, while rapamycin had a much less significant effect in control or FKBP5 knock down cells (Table S1). Addition of TCN could also sensitize paclitaxel in all four cell lines (Table S2). However, there was no significant difference in the degree of the sensitization effect between control and FKBP5 knockdown cell lines. LY294002 and rapamycin had limited effect on paclitaxel sensitization.

**Figure 3:**
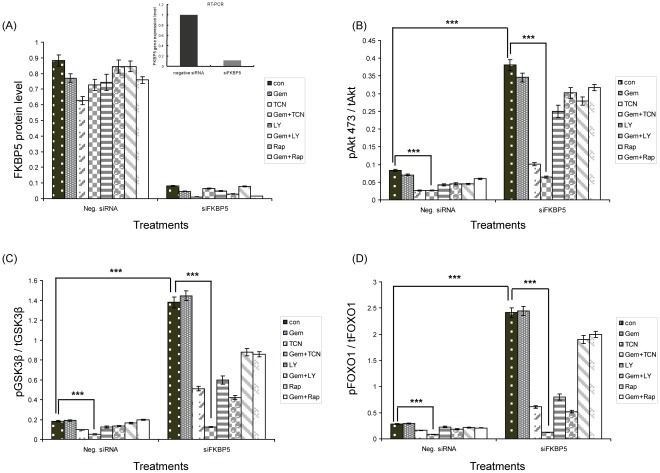
Effects of gemcitabine, TCN, LY294002, rapamycin and the combination of these agents on Akt signaling pathways in FKBP5 knock down pancreatic cancer cells. (A) FKBP5 knock down efficiency was determined by RT-PCR and Western blot. (B) SU86 cells were treated with DMSO (vehicle), 10 nM of gemcitabine alone, or 10 µM of TCN, 1.4 µM LY294002, and 1 nM rapamycin alone or in combination. Dose-dependent effects of different treatments on cellular viability were tested by MTS assay at 48 hours. Only combined treatment with TCN and gemcitabine inhibited Akt *in vitro*. ***P<0.005. (C and D) The combination of TCN and gemcitabine showed the most inhibition of pGSK3β and pFOXO1 when compared with other treatments. Statistical significance was assessed by *t* test and a p<0.005 was considered significant as shown by the asterisks (***).

The effects of LY294002, TCN and rapamycin in combination with gemcitabine on the Akt signaling pathway were also evaluated in SU86 cells. FKBP5 was knocked down using siRNA that targets FKBP5 (Figure 3A). Akt 473 phosphorylation was increased in FKBP5 knock down cells compared with control (Figure 3B, column 1 for both left and right panels, p<0.005), as well as downstream signaling molecules, such as phosphorylated GSK3β and FOXO1 (Figure 3C and D, column 1 for both left and right panels), consistent with our previous results [10]. TCN alone was sufficient to inhibit the Akt pathway as shown by decreased phosphorylation levels of Akt compared with control (Figure 3B, column 3 for both left and right panels, p<0.005), GSK3β and FOXO1 (Figure 3C and D, column 3 for both left and right panels). LY294002 also had an effect on the PI3K-Akt signaling pathway (Figure 3C and D, column 5 for both left and right panels). However, rapamycin alone had less of an inhibitory effect on PI3K-Akt pathway compared with TCN and LY294002 (Figure 3B, C and D, column 7 both left and right panels). TCN in combination with gemcitabine (Figure 3B, C and D, column 4 for both left and right panels,) further decreased the phosphorylation levels of Akt 473, GSK3β and FOXO1 when compared with either gemcitabine (Figure 3B, C and D, column 2 for both left and right panels) or TCN (Figure 3B, C and D, column 3 for both left and right panels) alone (p<0.005) and this effect was much more significant for TCN plus gemcitabine than for LY294002 or rapamycin plus gemcitabine (Figure 3B, C and D, column 6 and 8 for both left and right panels). Since knockdown of FKBP5 significantly increased Akt 473 phosphorylation levels, the reduction seen with TCN plus gemcitabine was much more significant in FKBP5 knockdown cells (Figure 3B, C and D, column 4 for both left and right panels), confirming our hypothesis that cells with low FKBP5 might depend more on Akt activation and, therefore, benefit more from the addition of Akt inhibitor.

### Enhanced Tumor Growth Inhibition with TCN Plus Gemcitabine *in vivo*

**Figure 4:**
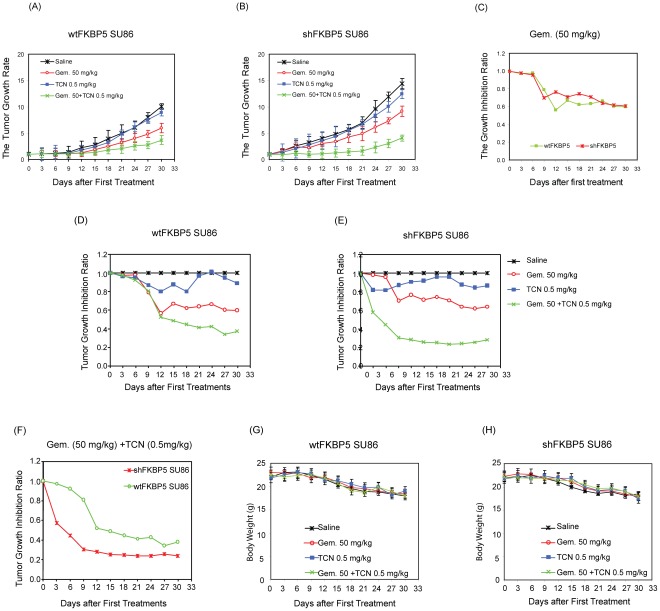
TCN sensitizes shFKBP5 pancreatic tumors to gemcitabine. Combination of TCN with gemcitabine effectively inhibited tumor growth *in vivo*. Mice with subcutaneously established tumors from wtFKBP5 SU86 or shFKBP5 SU86 cells entered the study when tumors reached ∼100 mm^3^ (day 0), and were treated with TCN at 0.5 mg/kg daily and/or gemcitabine at 50 mg/kg every 3 days for 30 days. (A) wtFKBP5 mice and (B) shFKBP5 mice tumor growth rate. ***P<0.005. (C) Comparison of maximal tumor suppression between wt and shFKBP5 xenografts. No significant difference in maximal suppression of tumor growth was observed for wt and shFKBP5 xenografts when treated with 50 mg/kg of gemcitabine alone. (D and E) Cotreatment with TCN significantly enhanced gemcitabine antitumor effect in both wt and shFKBP5 xenograft mice. **P<0.001; *P<0.05 (5 mice/group) as a comparison of tumor growth inhibition ratio between TCN plus gemcitabine and gemcitabine alone in wtFKBP5 and shFKBP5 groups, respectively. (F) Loss of FKBP5 expression results in increased sensitization effect for TCN on gemcitabine response. ***P<0.005 as a comparison of tumor growth inhibition ratio between wtFKBP5 and shFKBP5 mice. Mean ±SEM for five tumors at each data point. (G and H) Measurements of weight loss in wt and shFKBP5 xenografts. The toxic effects of administration of gemcitabine and gemcitabine plus TCN were determined by recording body weight for each mouse every 3 days throughout the experiment. ANOVA analysis was performed and p<0.005 was considered significant as shown by the asterisks (***).

Next, we used our xenograft mice with either wt or shFKBP5 SU86 cells to test whether FKBP5 knockdown mice might benefit more from the addition of the Akt inhibitor, TCN. Wild type and FKBP5 knockdown SU86 xenograft tumors were grown in nude mice. Once xenograft tumors formed, TCN and gemcitabine were injected i.p. A more rapid tumor growth rate was, once again, observed in shFKBP5 xenograft mice (Figure 4A and B). To evaluate antitumor efficacy, tumor-bearing mice were treated with TCN (0.5 mg/kg/day) i.p. for 4 weeks or gemcitabine (50 mg/kg) i.p. three times a week for 4 weeks in the presence or absence of TCN at 0.5 mg/kg, i.p. once a day for 5 days. Monotherapy with TCN alone was not effective in wt or FKBP5 knockdown xenografts, and there was no significant difference of maximal suppression of tumor growth in wt and shFKBP5 xenografts when treated with 50 mg/kg of gemcitabine alone (Figure 4C). However, cotreatment with TCN significantly enhanced gemcitabine antitumor effect compared with either gemcitabine or TCN alone in both wt and shFKBP5 xenograft mice (p<0.005 for both wt and shFKBP5) (Figure 4D and E). Greater inhibition effect of TCN plus gemcitabine was observed in shFBKP5 xenograft mice compared with wtFKBP5 (p<0.005) (Figure 4F). All treatments were well tolerated, and no animals died during the course of treatment. Therefore, the combination of gemcitabine and TCN showed a good safety profile in mice with no mortality or body weight loss (Figure 4G and H). Thus, the combination of TCN and gemcitabine exerted significantly greater *in vivo* antitumor effects than either agent alone, especially when the level of FKBP5 was decreased.

**Figure 5:**
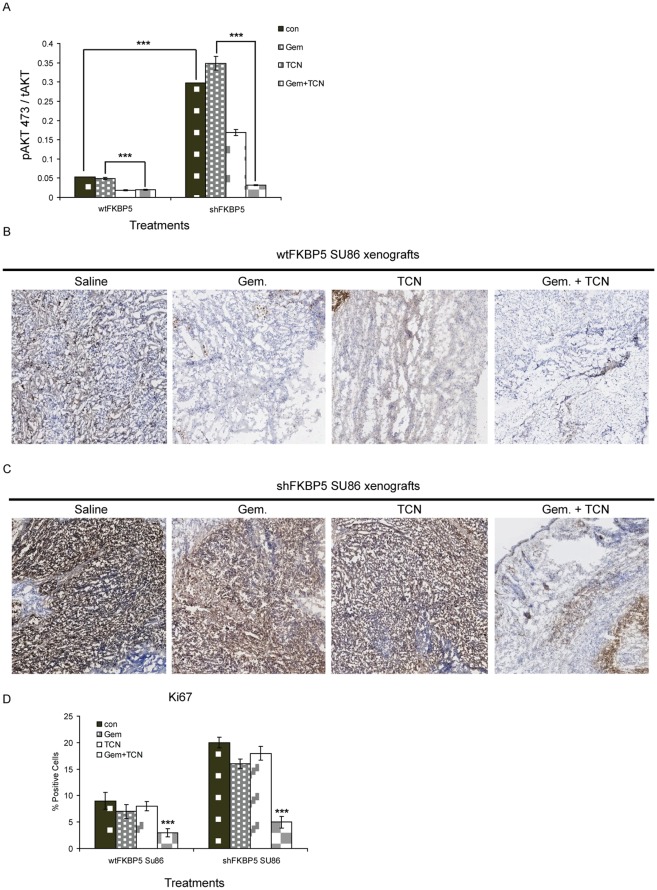
TCN and gemcitabine treatment decreases Akt activity and proliferation rate *in vivo*. (A) pAkt 473 levels were assayed in both wtFKBP5 and shFKBP5 tumor tissues. ***P<0.005. (B and C) Tumor samples from wtFKBP5 SU86 and shFKBP5 SU86 xenografts were prepared and stained for Ki67. (D) Quantification of Ki67 staining was expressed as percentage of positive cells (5 random fields for each sample, ***P<0.05). Statistical evaluation was performed with *t* test.

We next examined relative Akt 473 phosphorylation within the xenograft tumors after different treatments. We found that gemcitabine-resistant shFKBP5 xenografts had elevated levels of phosphorylated Akt 473 compared with wtFKBP5 (Figure 5A, column 1 for both left and right panels, p<0.005) as expected. TCN alone moderately inhibited phospho-Akt (53.1 ± 6.2% of control) (Figure 5A). Gemcitabine alone only slightly inhibited phospho-Akt in tumor (Figure 5A). With the addition of TCN, levels of phosphorylated Akt 473 were significantly reduced compared with controls (Figure 5A, p<0.005). To further address the underlying mechanism for inhibition of tumor progression, proliferation was determined by immunostaining in the xenograft tumors. Immunostaining of the proliferation marker Ki67 revealed more proliferating tumor cells in shFKBP5 xenografts when compared with controls (Figure 5B and C). The proliferative activity was lower in specimens treated with gemcitabine plus TCN than with gemcitabine alone in both wt and shFKBP5 xenografts (Figure 5D, p<0.01). These results strongly suggest that the combination of TCN and gemcitabine enhanced inhibition of the Akt pathway.

## Discussion

We recently reported that FKBP5 is a scaffolding protein that can enhance PHLPP-Akt interaction [10]. The functional consequence of this interaction results in negative regulation of Akt activity. Down regulation of FKBP5 results in decreased PHLPP-Akt interaction and increased Akt phosphorylation at the Ser473 site [10], suggesting that FKBP5 may function as a tumor suppressor, an important fact contributing to chemoresistance. Based on our previous findings with FKBP5 and its role in chemoresistance [9], [10], we tested this hypothesis *in vivo* using a xenograft mice model.

We found that tumors in shFKBP5 mice were more resistant to gemcitabine treatment and also exhibited a faster tumor growth rate (Figure 1A–D). This phenomenon appeared to involve the regulation of Akt activation, as determined by phosphorylated Akt and downstream signaling molecules (Figure 2). Since Akt is activated when FKBP5 is knocked down, we hypothesized that the addition of inhibitors targeting this pathway might reverse the drug resistance phenotype. The PI3K-Akt pathway has multiple drugable targets [25], [26], [27], [28], [29], [30], [31], so we tested a series of inhibitors targeting PI3K, Akt and mTOR. We observed different treatment effect in different cell lines (Tables 1, S1 and S2), which might be due to the cell or tissue specificity. We found that the specific Akt inhibitor, TCN, when administered together with gemcitabine had the best treatment outcome when compared with the other inhibitors tested (Table 1, and Figure S1), suggesting that the effect of FKBP5 on gemcitabine response depends mainly on Akt 473 phosphorylation. Consistent with the treatment outcomes, when we tested molecules within the Akt pathway that reflect Akt activation, treatment with LY294002 or rapamycin together with gemcitabine showed a less significant decrease of Akt activity when compared with gemcitabine plus TCN (Figure 3). As shown in Figure 4, even with wt xenografts, the combination of gemcitabine and TCN had a better tumor inhibition effect, suggesting that even in wt xenografts, Akt is hyperactivated and inhibition of this pathway could result in better treatment outcomes. However TCN showed a poor inhibition effect on proliferation when used as a single-agent in spite of the fact that it could reduce Akt phosphorylation, suggesting that other pathways also contribute to tumor development.

In addition to the role of FKBP5 in chemoresistance [10], based on our xenograft models it could also function as a tumor suppressor through negative regulation of the Akt pathway. As shown in Figures 3 and 5A, activity of the Akt pathway is significantly higher in FKBP5 knockdown SU86 xenografts than that in wild type SU86 xenografts and these observations correlated with higher tumor growth rates in shFKBP5 mice (Figure 1A). Therefore, probably because of the higher basal levels of Akt activity, shFKBP5 xenografts responded better to combination treatment, which was seen as enhanced inhibition of tumor growth (Figure 4F). This phenomenon was also reflected by decreased Akt 473 phosphorylation levels after gemcitabine and TCN treatment. The shFKBP5 xenografts showed a more dramatic decrease in Akt 473 phosphorylation levels wt xenografts (Figure 5A).

Our *in vivo* results further confirmed findings observed using the cell lines [11]. Those studies demonstrated that lack of expression of FKBP5 led to increased Akt phosphorylation at the regulatory S473 amino acid residue as well as for downstream genes in the Akt pathway such as phosphorylated FOXO1 and GSK3β. Therefore, FKBP5 could be a tumor suppressor in pancreatic cancer and it could also be a biomarker for response to chemotherapy, especially gemcitabine therapy, a first line treatment for pancreatic cancer. Our findings that a specific Akt inhibitor can reverse resistance to gemcitabine in FKBP5 knockdown cells and xenografts indicate that FKBP5 levels might be used to stratify patients into different treatment arms, such as gemcitabine or gemcitabine plus an Akt inhibitor. Future clinical studies will be needed to test this hypothesis. In addition, the mechanisms underlying differences between the effects of PI3K inhibition, mTOR inhibition and Akt inhibition in combination with gemcitabine need to be explored further. PI3K activation causes phosphatidylinositol-3,4,5-triphosphate (PIP3)-dependent membrane localization of Akt and PDK1, in which the latter can phosphorylate Akt 308 [32], [33], [34]. Therefore, the inhibition of PI3K might have less effect on 473 phosphorylation. Rapamycin can potentially activate Akt 473 phosphorylation in an mTOR-2 dependent manner due to relief of feedback inhibition of IGF-1R signaling [35]. That may explain why treatment with rapamycin plus gemcitabine failed to show a significant reduction of Akt 473 phosphorylation.

Obviously, these findings have to be confirmed by additional studies using human samples or transgenic mice. However, currently it is challenging to obtain adequate clinical samples with similar clinical characteristics treated with gemcitabine alone to determine the relationship between FKBP5 and treatment response since most patients are treated with multiple agents. Certainly future clinical trials designed to test the effect of this biomarker will be essential to determine whether FKBP5 can be used as a biomarker for the selection of treatment for individual patients.

In summary, the findings presented here indicated the importance of FKBP5 in pancreatic tumor growth and chemoresistance. Moreover, the data suggest that specific Akt inhibitors might be promising adjuvant therapies for pancreatic cancer, especially in patients with lower level of FKBP5. These findings could help individualize therapy to achieve better treatment outcomes for pancreatic cancer patients.

## Supporting Information

Figure S1 **TCN sensitizes FKBP5−/− human pancreatic and breast cancer cells to gemcitabine** ***in vitro*** **.** (A) Knockdown efficiency for FKBP5 in BXPC3, ASPC1, MCF7 or HS578T cells determined by real-time QRT-PCR. (B)-(F). Cytotoxicity was determined with MTS assays in BXPC3, ASPC1, SU86, MCF7, and HS578T cells. Cells were treated with vehicle (DMSO), various concentrations of gemcitabine (0.01, 0.1, 1, 5, 10, 25, 50, 100, and 500 nM) alone or in combination with 10 µM of TCN, 1.4 µM LY294002, or 1 nM rapamysin. Each data point is mean for 3 independent experiments. Error bars indicate standard error of the mean (SEM).
(PDF)

Figure S2 **Cytotoxicity of TCN, LY294002 and rapamycin in human pancreatic and breast cancer cells.** (A) Knockdown efficiency for FKBP5 in BXPC3, ASPC1, MCF7 or HS578T cells determined by real-time QRT-PCR. (B)-(D) BXPC3, ASPC1, MCF7 or HS578T cells were plated in 96-well plates, treated for 48 hours with various concentrations of TCN (0.1, 0.5, 1, 2.5, 5, 10, 25, 50, and 100 µM), LY294002 (0.1, 0.5, 1, 2.5, 5, 10, 25, 50, and 100 µM) and rapamycin (0.1, 0.5, 1, 2.5, 5, 10, 25, 50, and 100 nM), followed by MTS assay as described under Methods. Each data point is an average of triplicates from 3 independent experiments. Error bars indicate standard error of the mean (SEM).
(PDF)

Table S1 **Combinatory effects of etoposide and inhibitors targeting PI3K-Akt-mTOR pathway in human pancreatic and breast cancer cells.**
(PDF)

Table S2 **Combinatory effects of paclitaxel and inhibitors targeting PI3K-Akt-mTOR pathway in human pancreatic and breast cancer cells.**
(PDF)
