# Supplementary figures and images for: FKBP5 as a Selection Biomarker for Gemcitabine and Akt Inhibitors in Treatment of Pancreatic Cancer
Source: PLoS One. 2012 May 9;7(5):e36252. doi: 10.1371/journal.pone.0036252 (PMC3348935; doi:10.1371/journal.pone.0036252)

Figure S1

(A)

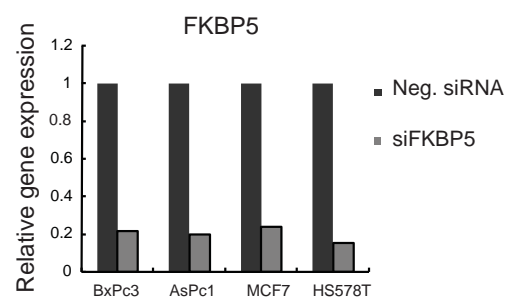

(B)

BXPC3

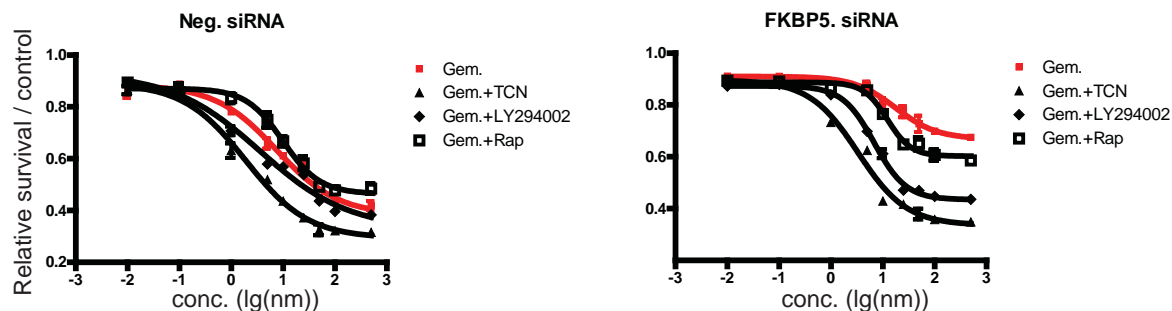

(C)

ASPC1

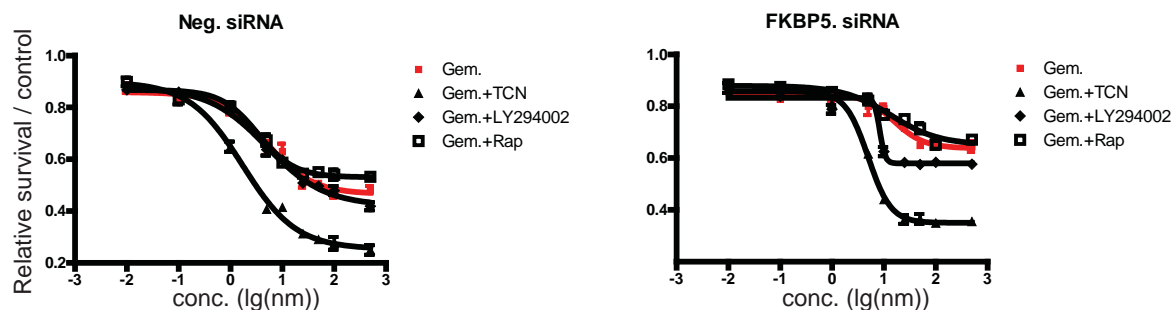

(D)

SU86

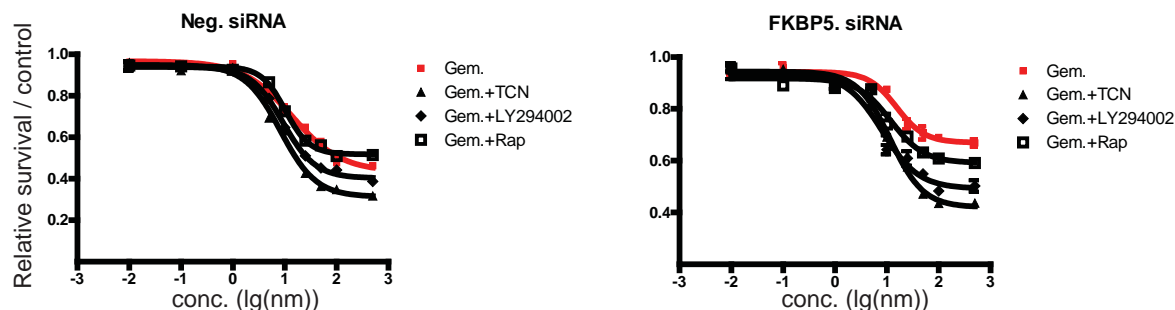

(E)

MCF7

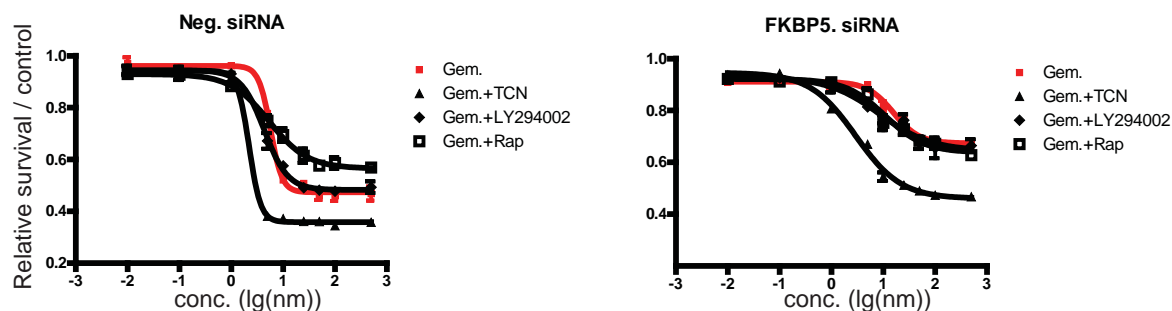

(F)

HS578T

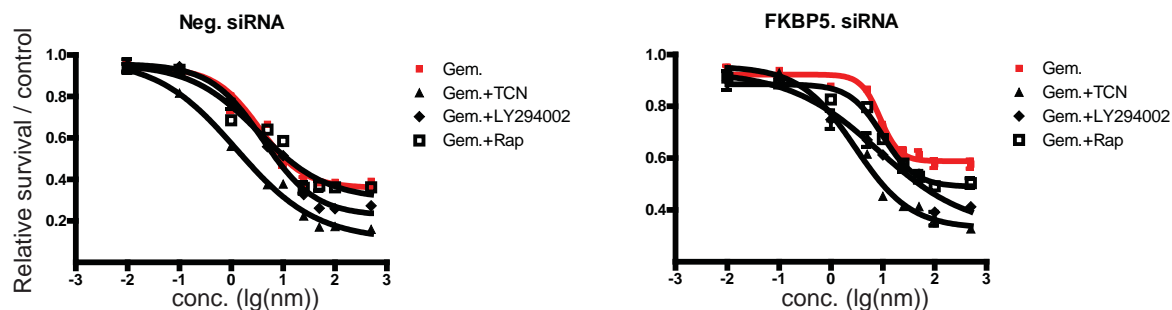

Supplement: Figure S1 — TCN sensitizes FKBP5−/− human pancreatic and breast cancer cells to gemcitabine in vitro . (A) Knockdown efficiency for FKBP5 in BXPC3, ASPC1, MCF7 or HS578T cells determined by real-time QRT-PCR. (B)-(F). Cytotoxicity was determined with MTS assays in BXPC3, ASPC1, SU86, MCF7, and HS578T cells. Cells were treated with vehicle (DMSO), various concentrations of gemcitabine (0.01, 0.1, 1, 5, 10, 25, 50, 100, and 500 nM) alone or in combination with 10 µM of TCN, 1.4 µM LY294002, or 1 nM rapamysin. Each data point is mean for 3 independent experiments. Error bars indicate standard error of the mean (SEM). (PDF) [file pone.0036252.s001.pdf]

Figure S2

(A)

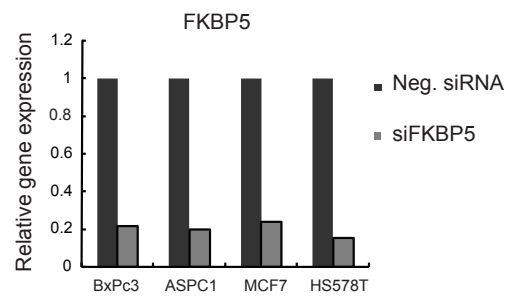

(B)

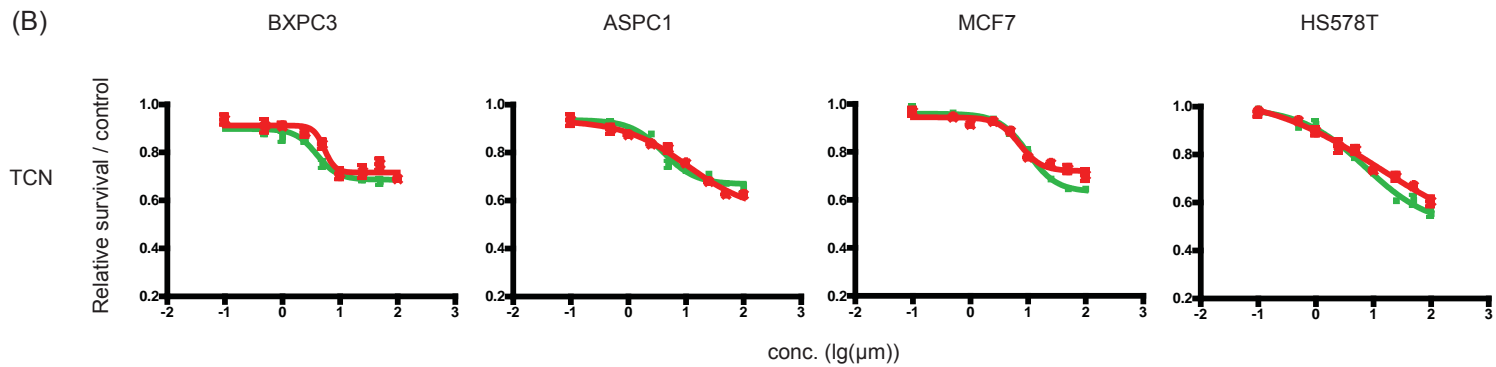

(C)

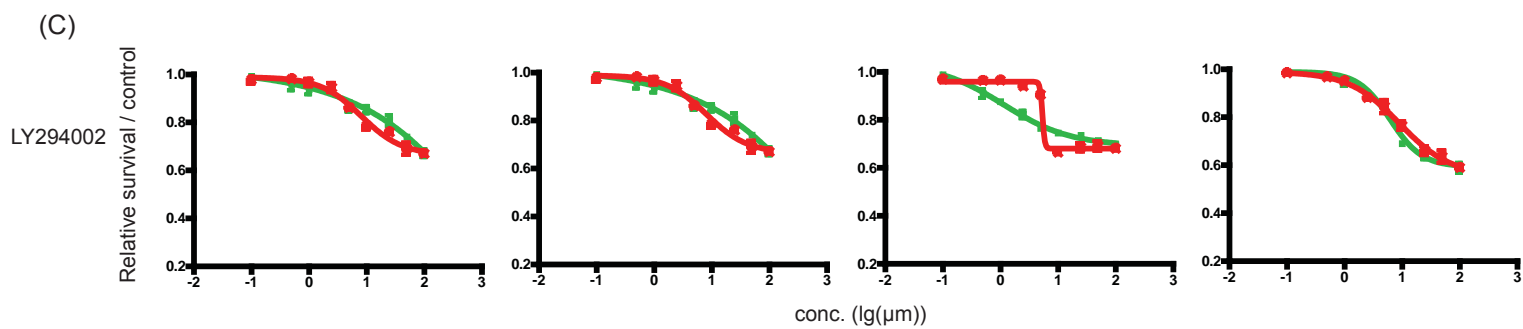

(D)

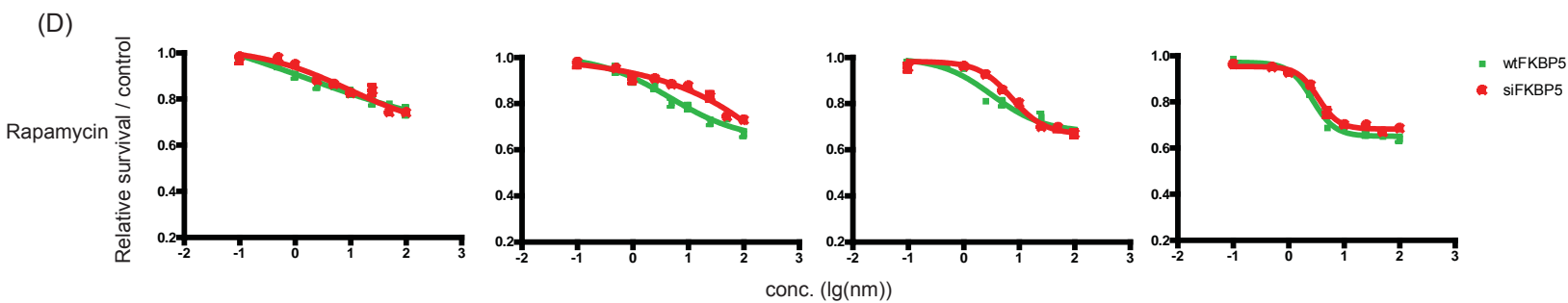

Supplement: Figure S2 — Cytotoxicity of TCN, LY294002 and rapamycin in human pancreatic and breast cancer cells. (A) Knockdown efficiency for FKBP5 in BXPC3, ASPC1, MCF7 or HS578T cells determined by real-time QRT-PCR. (B)-(D) BXPC3, ASPC1, MCF7 or HS578T cells were plated in 96-well plates, treated for 48 hours with various concentrations of TCN (0.1, 0.5, 1, 2.5, 5, 10, 25, 50, and 100 µM), LY294002 (0.1, 0.5, 1, 2.5, 5, 10, 25, 50, and 100 µM) and rapamycin (0.1, 0.5, 1, 2.5, 5, 10, 25, 50, and 100 nM), followed by MTS assay as described under Methods. Each data point is an average of triplicates from 3 independent experiments. Error bars indicate standard error of the mean (SEM). (PDF) [file pone.0036252.s002.pdf]
