# Supplementary material for: FKBP5 as a Selection Biomarker for Gemcitabine and Akt Inhibitors in Treatment of Pancreatic Cancer
Source: PLoS One. 2012 May 9;7(5):e36252. doi: 10.1371/journal.pone.0036252 (PMC3348935; doi:10.1371/journal.pone.0036252)
Supplement: Table S1 — Combinatory effects of etoposide and inhibitors targeting PI3K-Akt-mTOR pathway in human pancreatic and breast cancer cells. (PDF) [file pone.0036252.s003.pdf]

Table S1. Combinatory effects of etoposide and inhibitors targeting PI3K-Akt-mTOR pathway in human pancreatic and breast cancer cells.

**Cells**	**Agent**	**IC_{50} (μM)[a]**		**p value[b]**	
		Neg. siRNA	siFKBP5	Neg. siRNA	siFKBP5
BXPC3	Eto	0.806	0.5229		
	Eto+TCN	0.535	0.4616	0.0005	0.0003
	Eto+LY294002	0.5586	0.5418	0.0023	0.001
	Eto+Rap	2.503	2.371	0.1509	0.1755
ASPC1	Eto	5.109	12.1		
	Eto+TCN	4.125	0.2513	0.001	0.0007
	Eto+LY294002	3.822	4.252	0.1985	0.0216
	Eto+Rap	24.16	10.96	0.0171	0.2744
MCF7	Eto	0.9081	4.953		
	Eto+TCN	0.498	1.774	0.0036	0.0003
	Eto+LY294002	0.6957	2.472	0.0045	0.0054
	Eto+Rap	1.468	5.026	0.9096	0.0769
HS578T	Eto	0.9653	1.651		
	Eto+TCN	0.4204	0.7752	0.003	0.001
	Eto+LY294002	0.6424	0.9473	0.3412	0.0099
	Eto+Rap	0.6865	1.215	0.931	0.0342

[a] The values represent the average of three independent experiments.

[b] IC50 values between combination treatment vs. Eto along were analyzed statistically by performing t tests

Abbreviations: Eto, etoposide; TCN, tricirbine; Rap, rapamycin
