# Supplementary material for: FKBP5 as a Selection Biomarker for Gemcitabine and Akt Inhibitors in Treatment of Pancreatic Cancer
Source: PLoS One. 2012 May 9;7(5):e36252. doi: 10.1371/journal.pone.0036252 (PMC3348935; doi:10.1371/journal.pone.0036252)
Supplement: Table S2 — Combinatory effects of paclitaxel and inhibitors targeting PI3K-Akt-mTOR pathway in human pancreatic and breast cancer cells. (PDF) [file pone.0036252.s004.pdf]

Table S2. Combinatory effects of paclitaxel and inhibitors targeting PI3K-Akt-mTOR pathway in human pancreatic and breast cancer cells.

**Cells**	**Agent**	**IC_{50} (μM)**[a]		**p value**[b]	
		Neg. siRNA	siFKBP5	Neg. siRNA	siFKBP5
BXPC3	Tax	0.01033	0.02604		
	Tax+TCN	0.007907	0.01524	0.005	0.004
	Tax+LY294002	0.01149	0.0247	0.8405	0.7542
	Tax+Rap	0.01495	0.02059	0.9634	0.3702
ASPC1	Tax	0.003702	0.008071		
	Tax+TCN	0.001794	0.004039	0.0071	0.005
	Tax+LY294002	0.002507	0.00666	0.006	0.1042
	Tax+Rap	0.004582	0.005431	0.1154	0.005
MCF7	Tax	0.009508	0.01571		
	Tax+TCN	0.004629	0.005816	0.0046	0.0003
	Tax+LY294002	0.007369	0.009197	0.0045	0.0054
	Tax+Rap	0.006446	0.00876	0.9096	0.0769
HS578T	Tax	0.01019	0.02712		
	Tax+TCN	0.00696	0.01014	0.007	0.006
	Tax+LY294002	0.01133	0.01897	0.2457	0.0157
	Tax+Rap	0.009164	0.01654	0.6941	0.006

[a] The values represent the average of three independent experiments.

[b] IC50 values between combination treatment vs. Tax along were analyzed statistically by performing t tests

Abbreviations: Tax, paclitaxel; TCN, tricirbine; Rap, rapamycin
